# Intratracheal budesonide mixed with surfactant to increase survival free of bronchopulmonary dysplasia in extremely preterm infants: study protocol for the international, multicenter, randomized PLUSS trial

**DOI:** 10.1186/s13063-023-07257-5

**Published:** 2023-05-09

**Authors:** Brett J. Manley, C. Omar F. Kamlin, Susan Donath, Li Huang, Pita Birch, Jeanie L. Y. Cheong, Peter A. Dargaville, Jennifer A. Dawson, Lex W. Doyle, Susan E. Jacobs, Rodney Wilson, Peter G. Davis, Christopher J. D. McKinlay

**Affiliations:** 1 The Royal Women’s Hospital, Department of Obstetrics and Gynaecology, The University of Melbourne, Murdoch Children’s Research Institute, Melbourne, Australia; 2 Department of Paediatrics, Murdoch Children’s Research Institute, the University of Melbourne, Melbourne, Australia; 3 The University of Melbourne, Melbourne, Australia; 4 Department of Neonatology, Mater Mother’s Hospitals South Brisbane, Brisbane, Australia; 5 Royal Hobart Hospital, Hobart, Australia; 6 Menzies Institute for Medical Research, University of Tasmania, Hobart, Australia; 7 Consumer Advisor, Melbourne, Australia; 8 Department of Paediatrics: Child and Youth Health, the University of Auckland, Kidz First Neonatal Care, TeWhatu Ora Counties Manukau, Auckland, New Zealand

**Keywords:** Infant, Extremely preterm, Bronchopulmonary dysplasia, Respiratory distress syndrome, Neonatal intensive care, Pulmonary surfactant, Postnatal corticosteroids, Budesonide, Intratracheal, Neonatal mortality

## Abstract

**Background:**

Bronchopulmonary dysplasia (BPD), an inflammatory-mediated chronic lung disease, is common in extremely preterm infants born before 28 weeks’ gestation and is associated with an increased risk of adverse neurodevelopmental and respiratory outcomes in childhood. Effective and safe prophylactic therapies for BPD are urgently required. Systemic corticosteroids reduce rates of BPD in the short-term but are associated with poorer neurodevelopmental outcomes if given to ventilated infants in the first week after birth. Intratracheal administration of corticosteroid admixed with exogenous surfactant could overcome these concerns by minimizing systemic sequelae. Several small, randomized trials have found intratracheal budesonide in a surfactant vehicle to be a promising therapy to increase survival free of BPD.

**Methods:**

An international, multicenter, double-blinded, randomized trial of intratracheal budesonide (a corticosteroid) mixed with surfactant for extremely preterm infants to increase survival free of BPD at 36 weeks’ postmenstrual age (PMA; primary outcome). Extremely preterm infants aged < 48 h after birth are eligible if: (1) they are mechanically ventilated, or (2) they are receiving non-invasive respiratory support and there is a clinical decision to treat with surfactant. The intervention is budesonide (0.25 mg/kg) mixed with poractant alfa (200 mg/kg first intervention, 100 mg/kg if second intervention), administered intratracheally via an endotracheal tube or thin catheter. The comparator is poractant alfa alone (at the same doses). Secondary outcomes include the components of the primary outcome (death, BPD prior to or at 36 weeks’ PMA), potential systemic side effects of corticosteroids, cost-effectiveness, early childhood health until 2 years of age, and neurodevelopmental outcomes at 2 years of age (corrected for prematurity).

**Discussion:**

Combining budesonide with surfactant for intratracheal administration is a simple intervention that may reduce BPD in extremely preterm infants and translate into health benefits in later childhood. The PLUSS trial is powered for the primary outcome and will address gaps in the evidence due to its pragmatic and inclusive design, targeting all extremely preterm infants regardless of their initial mode of respiratory support. Should intratracheal budesonide mixed with surfactant increase survival free of BPD, without severe adverse effects, this readily available intervention could be introduced immediately into clinical practice.

**Trial registration:**

Australian New Zealand Clinical Trials Registry (https://www.anzctr.org.au), ACTRN12617000322336. First registered on 28th February 2017.

## Administrative information

**Table:** 

Title {1}	Intratracheal budesonide mixed with surfactant to increase survival free of bronchopulmonary dysplasia in extremely preterm infants: study protocol for the international, multicenter, randomized PLUSS trial
Trial registration {2}	Australian New Zealand Clinical Trials Registry; ACTRN12617000322336. Prospectively registered on 28^th^ February 2017
Protocol version {3}	Version 8: 14^th^ July 2020 (or any subsequent amended protocols)
Funding {4}	1. National Health and Medical Research Council (NHMRC), Australia (Grant No. 1158555) 2. Investigator-initiated grant from *Chiesi Farmaceutici*, Parma, Italy, to reimburse centers for the cost of poractant alfa used in the trial 3. Thrasher Research Fund, USA: “E.W. Thrasher Award” to fund longer-term outcome assessments at 2 years 4. Cure Kids New Zealand (Grant No. 3614) to help fund longer-term outcome assessments at 2 years in New Zealand
Author details {5a}	BJM, COFK, PGD, JLYC, LWD, JAD, SEJ: The Royal Women’s Hospital, Melbourne, Australia; Department of Obstetrics and Gynaecology, The University of Melbourne, Melbourne, Australia; Murdoch Children’s Research Institute, Melbourne, Australia CJDM: Department of Paediatrics: Child and Youth Health, the University of Auckland, Auckland, New Zealand; Kidz First Neonatal Care, Te Whatu Ora Counties Manukau, New Zealand PAD: Royal Hobart Hospital, Hobart, Australia; Menzies Institute for Medical Research, University of Tasmania, Hobart, Australia PB: Department of Neonatology, Mater Mother’s Hospitals South Brisbane, Brisbane, Australia SD: Murdoch Children’s Research Institute, Melbourne, Australia; Department of Paediatrics, the University of Melbourne, Melbourne, Australia LH: The University of Melbourne, Melbourne, Australia
Name and contact information for the trial sponsor {5b}	Melbourne Children’s Trials Centre, Murdoch Children’s Research Institute (MCRI), Melbourne, Australia (mctc@mcri.edu.au)
Role of sponsor {5c}	The trial Sponsor is responsible for the initiation, management and financing (or arranging the financing) of the trial and carries the medicolegal responsibility associated with its conduct The Sponsor is also responsible for ensuring that appropriate approvals are obtained prior to the commencement of the clinical trial, that conditions of any approvals are adhered to during the course of the clinical trial and ensuring that the ethics principles of research merit and integrity, justice, beneficence and respect are applied to the conduct of clinical trials. The Sponsor also ensures that the trial is appropriately monitored for compliance with the protocol The Trial Steering Committee, chaired by BJM, is responsible for the study design, management and analysis of data, and decision to publish the results

## Introduction

### Background and rationale {6a}

#### Burden of Illness

Prematurity remains the greatest cause of neonatal mortality and morbidity worldwide. Infants born extremely preterm (< 28 weeks’ gestation) are at greatest risk. Approximately 1,100 infants born extremely preterm are cared for in neonatal intensive care units (NICUs) in Australia and New Zealand annually [1], and 780,000 worldwide [2]. With modern intensive care, around 85% of infants born extremely preterm survive, but at least half develop bronchopulmonary dysplasia (BPD) [1], the chronic lung disease of prematurity. BPD is independently associated with increased infant mortality, subsequent hospitalization with respiratory illness, and adverse neurodevelopmental outcomes [3–6]. There is evidence of worsening lung function in surviving Australian children over time [7], and infants with BPD are at risk of developing early chronic obstructive pulmonary disease as adults [8, 9].

BPD is traditionally diagnosed if an infant is receiving respiratory support or supplemental oxygen at 36 weeks’ post-menstrual age (PMA). It is most common in infants born extremely preterm who are exposed to prolonged mechanical ventilation and oxygen therapy for respiratory distress syndrome (RDS), the preterm lung disease associated with surfactant deficiency. Almost all infants born extremely preterm have RDS, and about 90% are treated with exogenous surfactant therapy in the first 24 h after birth [1]. Surfactant has been available since the 1990s and is a safe and effective therapy for RDS, administered directly into the trachea via an endotracheal tube or thin catheter. Despite the advent of surfactant therapy and other major advances in neonatal care, BPD rates are static [10], and there are very few therapies that safely reduce BPD rates. Therefore, prevention of BPD is a major research priority [11].

#### Systemic corticosteroids effectively treat BPD but are associated with harm

Inflammation is the primary mediator of the pathogenesis of BPD [12]. Anti-inflammatory therapies such as postnatal corticosteroids have long been the focus of preventive interventions. However, there has been significant harm associated with their *systemic* (enteral/intravenous) administration in preterm infants, especially with higher doses in the first week after birth [13]. Short-term systemic adverse effects include hyperglycemia, sepsis, hypertension, cardiomyopathy, gastrointestinal hemorrhage, intestinal perforation, and reduced growth. Long-term systemic adverse effects include neurodevelopmental delay and cerebral palsy [13].

In the PREMILOC trial, administration of prophylactic low-dose systemic hydrocortisone, compared with placebo, in extremely preterm infants for the first 10 days after birth increased survival without BPD at 36 weeks’ PMA (60% vs. 51%, OR = 1.48, 95% CI 1.02 to 2.16), with no adverse effects on neurodevelopment at 22 months’ corrected age [14, 15]. However, prophylactic systemic hydrocortisone has not been widely adopted due to several concerns: the trial was stopped early (66% of planned sample size); among infants born at < 26 weeks’ gestation, those exposed to hydrocortisone had a two-fold increase in late-onset sepsis; and overall morbidity was high, including prolonged ventilation, suggesting results may not be generalizable. Therefore, in many centers, systemic corticosteroids are currently reserved for *later treatment* of the sickest, mechanical ventilator-dependent extremely preterm infants, in whom the benefits may outweigh the risks [16].

#### Inhaled corticosteroids are not the solution

Early trials of inhaled corticosteroids in extremely preterm infants did not show benefit for reducing BPD [17], possibly due to technical difficulties with delivery of the drug to the distal airway. More recently the NEUROSIS trial showed that administration of inhaled budesonide, compared with placebo, in extremely preterm infants from day one until discontinuation of supplemental oxygen and respiratory support, or until 32 weeks’ PMA, may increase survival without BPD (60% vs. 54%, RR = 0.86, 95% CI 0.75 to 1.00) [18]. However, inhaled budesonide therapy increased mortality at 18 to 22 months (20% vs. 15%, RR = 1.37, 95% CI 1.01 to 1.86) [19]. Notably, in this trial, infants were treated for an average of 39 days, with a very high average cumulative budesonide dose of 21 mg; the most immature infants received ≥ 30 mg. Given the adverse outcomes with little clear benefit, clinicians have not embraced this therapy.

#### Intratracheal budesonide mixed with surfactant is a highly promising therapy

A long-acting anti-inflammatory agent administered during the acute phase of RDS, combined with a safe vehicle to deliver it effectively to the distal airways, has great potential to modulate the inflammatory pathway to BPD, and minimize systemic side-effects. Budesonide, compared with other corticosteroids, has a greater affinity for the glucocorticoid receptor and a higher ratio of topical to systemic anti-inflammatory activity [20]. Intratracheal budesonide inhibits pro-inflammatory cytokines and increases the production of anti-inflammatory cytokines [21, 22]. Unlike other commonly-used corticosteroids in inflammatory airway disease, budesonide reversibly conjugates with intracellular fatty acids in lung tissue to form a lipophilic ester within an hour of intratracheal instillation. These esters are slowly hydrolyzed to release free intracellular budesonide, prolonging its local anti-inflammatory activity for up to 1 week [23, 24].

Intratracheal budesonide has a favorable safety profile, with minimal systemic absorption. Because of the depot effect created by esterification within the epithelium, very little budesonide (< 10%) is absorbed into the systemic circulation [21, 24]. Furthermore, free plasma budesonide is rapidly metabolized and inactivated by the liver, predominantly to 16α-hydroxyprednisolone, a metabolite with low glucocorticoid activity, further minimizing any systemic effects [25, 26]. Importantly, as a safety endpoint, budesonide was undetectable in the brain following intratracheal administration in a preterm lamb model [27].

Surfactant effectively delivers budesonide to the distal airways. In vitro studies have shown that adding budesonide to surfactant promotes diffusion of budesonide along an air–liquid interface, without adversely affecting surfactant performance [28–30]. Similarly, in animal studies, admixture of budesonide in surfactant, compared with saline, resulted in better alveolar distribution of budesonide [31], a longer half-life in the lung compared with budesonide alone [32], and a reduction in lung inflammation without adversely affecting the physiological response to surfactant treatment [30]. Moreover, intratracheal instillation of budesonide with surfactant in a rabbit RDS model increased alveolar area, decreased alveolar wall thickness, and increased surfactant production, suggesting that budesonide also promotes lung maturation in a manner similar to antenatal corticosteroids [33]. Thus, the combination of budesonide and surfactant in pre-clinical studies appears to be superior to either budesonide or surfactant alone.

There are promising data from previous clinical trials of intratracheal budesonide. In a pilot trial (*N* = 116), Yeh et al. found that intratracheal budesonide mixed with surfactant (beractant) compared with surfactant alone, increased survival without BPD in infants with birth weight < 1500 g [24], with no adverse effects on neurodevelopment at 2 to 3 years corrected age [34]. The same group subsequently undertook a three-center trial in Taiwan and the USA, randomizing infants (*N* = 265) with “severe” RDS, defined as receiving mechanical ventilation with fraction of inspired oxygen (FiO_2_) > 0.50 within 4 h of birth, to surfactant (100 mg/kg beractant) plus budesonide (0.25 mg/kg) or surfactant alone, repeated every 8 h until the infant had FiO_2_ < 0.30 or was extubated from mechanical ventilation [32]. Infants could receive up to 6 treatments, though most received one or two. The surfactant plus budesonide group had a lower incidence of death or BPD (42% vs. 66%, RR = 0.58, 95% CI 0.44 to 0.78), fewer recurrent upper respiratory tract infections in infancy, and a possible reduction in neurodevelopmental impairment at 2 to 3 years of age (31% vs. 39%). The incidence of potential systemic corticosteroid side effects (e.g., hypertension, hyperglycemia, sepsis) did not differ between groups. Importantly, budesonide not only reduced BPD but also improved short-term respiratory function as measured by lower FiO_2_ and fewer doses of surfactant, suggesting a synergistic effect with surfactant for the treatment of RDS. Infants who received intratracheal budesonide had lower levels of pro-inflammatory interleukins in tracheal aspirates throughout the first week [32]. There was no significant difference in mortality (13% vs. 16%, RR = 0.96, 95% CI 0.87 to 1.06). Two additional, small, randomized trials have been published (total of 250 preterm infants enrolled across both trials) that have shown promising effects of intratracheal budesonide mixed with surfactant [35, 36]. However, none of the published trials definitively answer the question of whether intratracheal budesonide mixed with surfactant improves important clinical outcomes for extremely preterm infants, and thus, clinical practice has not changed.

#### The PLUSS trial of intratracheal budesonide will address the critical gaps in evidence

We present the Preventing Lung disease Using Surfactant and Steroid (PLUSS) trial, a double-blind, randomized trial of intratracheal budesonide mixed with surfactant in extremely preterm infants to increase survival without BPD. The PLUSS trial will address the following limitations of design and interpretation associated with previous studies:

##### Generalizability

The trial by Yeh et al. targeted high-risk mechanically ventilated extremely preterm infants [32]. These infants are increasingly being managed using ‘non-invasive’ respiratory support, such as continuous positive airway pressure (CPAP) to avoid or limit exposure to barotrauma and volutrauma from mechanical ventilation. PLUSS will determine whether intratracheal budesonide is effective in a broader group of infants born extremely preterm, regardless of their RDS severity, mode of respiratory support, technique of surfactant administration, or previous surfactant treatment.

##### Sample and effect size

The treatment effect demonstrated by Yeh et al. [32] requires confirmation in a larger trial. An editorial accompanying the Yeh publication states that the findings need to be replicated, including in infants with less severe RDS [37]. Even an effect on BPD half the magnitude of that reported would represent a great advance in BPD prevention.

##### Type of surfactant

Applicability of the study by Yeh et al. [32] is further limited by the use of bovine surfactant which requires a larger instillation volume (4 mL/kg) and is much less commonly used in Australasia and Europe than the preferred porcine-derived surfactant, poractant alfa (Curosurf®, *Chiesi Farmaceutici*, Parma, Italy), which is used in the PLUSS trial [1, 38]. As the volume instilled is smaller (1.25–2.5 mL/kg), poractant alfa can be more readily applied as a drug delivery vehicle in a population of infants receiving surfactant therapy for RDS managed with non-invasive respiratory support and using less invasive surfactant administration techniques [39, 40].

#### Significance

Exogenous surfactant is a proven effective therapy for RDS in preterm infants. Combining budesonide with surfactant is a simple intervention that may increase survival free of BPD in the highest-risk population of extremely preterm infants. Previous studies of intratracheal budesonide mixed with surfactant in mechanically ventilated infants provide preliminary evidence of feasibility, safety, and potential benefit [32, 35, 36]. The pragmatic and inclusive design of the PLUSS trial, targeting all extremely preterm infants with RDS, regardless of their initial mode of respiratory support, will mean the results are applicable worldwide. Should intratracheal budesonide mixed with surfactant reduce BPD, without adverse effects, this readily available and inexpensive intervention could be introduced immediately into routine clinical practice, with potential to have a lifelong impact on health outcomes and significantly reduce burden on healthcare resources.

### Objectives {7}

#### Aim

To determine whether intratracheal budesonide mixed with surfactant increases survival without BPD in infants born before 28 weeks’ gestation.

#### Hypotheses

Early administration of intratracheal budesonide mixed with a surfactant to extremely preterm infants will increase survival free of BPD at 36 weeks’ PMA.

##### Secondary hypotheses

The intervention will *not* be associated with short-term adverse corticosteroid effects and will be safe and improve outcomes to 2 years of age (corrected for prematurity).

### Trial design {8}

An international, multicenter, two-arm, parallel, double-blind, superiority randomized controlled trial.

## Methods: participants, interventions, and outcomes

### Study setting {9}

An international, multicenter randomized trial in neonatal intensive care units (NICUs) in tertiary level perinatal centers conducted at the following 21 participating sites (at the time this protocol was submitted for publication):

- Australia:
  - o Victoria: The Royal Women’s Hospital, Monash Children’s Hospital, Mercy Hospital for Women
  - o New South Wales: Royal Hospital for Women, Royal North Shore Hospital, John Hunter Hospital, Royal Prince Alfred Hospital
  - o Queensland: Royal Brisbane and Women’s Hospital, Mater Mothers’ Hospital
  - o South Australia: Flinders Medical Centre, Women’s and Children’s Hospital
  - o Western Australia: King Edward Memorial Hospital
  - o Australian Capital Territory: Canberra Hospital
  - o Tasmania: Royal Hobart Hospital
- New Zealand:
  - o Kidz First Hospital (Counties Manukau, Auckland)
  - o National Women’s Health (Auckland)
  - o Wellington Regional Hospital
  - o Christchurch Hospital
  - o Waikato Hospital
- Canada:
  - o Royal Alexandra Hospital, Edmonton
- Singapore:
  - o KK Women’s and Children’s Hospital

### Eligibility criteria {10}

Inclusion criteria (all must be satisfied):

1. Born before 28 weeks’ gestation
2. Less than 48 h of age
3. No more than 1 prior dose of exogenous surfactant administered
4. Receiving:
  - mechanical ventilation via an endotracheal tube, regardless of ventilation settings or oxygen requirement (automatically qualify for the intervention) or
  - non-invasive respiratory support (any type including continuous positive airway pressure [40], nasal intermittent positive pressure ventilation [NIPPV], or nasal high flow) *and* there is a clinical decision to treat with surfactant (first or second dose
5. Prospective, written, informed parental/guardian consent obtained

Exclusion criteria (any one or more mandates exclusion):

1. More than one prior surfactant dose
2. Prior treatment with postnatal corticosteroids for the prevention of lung disease (inhaled, nebulized, intratracheal, or systemic)
3. The infant is considered unlikely to survive the immediate postnatal transition and/or is not going to be admitted to the NICU
4. Known or suspected major congenital anomaly that is likely to affect respiratory status including a postnatal clinical diagnosis of severe pulmonary hypoplasia following premature prolonged rupture of fetal membranes with resultant severe oligo- or anhydramnios, where the clinician feels survival is unlikely
5. The infant is likely to be transferred to a non-participating NICU within 24 h of birth.

### Who will take informed consent? {26a}

Informed parental/guardian consent will be obtained prior to randomization by a clinician or researcher trained to obtain consent for the trial. Consent will be obtained either antenatally or postnatally. In all cases, written consent will be obtained using a specifically designed Participant Information and Consent Form (PICF) which may be modified to meet the requirements of each participating center’s human research ethics committee (HREC). A copy of the PICF will be provided to the parents and documented in the infant’s hospital record. Parent(s)/guardian(s) are free to withdraw their infant from the study at any time. Permission will be sought from families who withdraw their infant from the study to allow researchers to continue data collection from their child’s hospital record.

### Additional consent provisions for collection and use of participant data and biological specimens {26b}

In all Australian centers, separate consent will be obtained for Medicare data linkage using Department of Health consent forms to enable follow-up of study participants’ use of pharmaceuticals and medical services until 2 years’ corrected age. In other countries, consent will be sought for equivalent data using the appropriate methods.

Two sub-studies have been designed to run in tandem with the main trial, *but will have separate trial protocols and be reported separately*:

1. In select centers, infants will be enrolled in a pharmacokinetic sub-study with the aim of testing the hypothesis that systemic budesonide uptake will be minimal, and rapidly cleared from the infant’s circulation, without any significant effect on endogenous plasma glucocorticoid activity.
2. “PLUSS-HEARTS”: In select centers with access to dedicated neonatal echocardiographic services, a sub-study will be conducted to evaluate the effect of intratracheal budesonide combined with surfactant on the patency of the ductus arteriosus at 48–72 h of age.

## Interventions

### Explanation for the choice of comparators {6b}

Exogenous surfactant is the current standard of care for RDS in preterm infants. Natural surfactants include products of bovine and porcine origin, with the latter the most commonly used in Australasia and Europe, and increasingly around the world. Poractant alfa (Curosurf®, *Chiesi Farmaceutici*, Italy) is a porcine surfactant with an advantage over bovine (beractant, Survanta®, Abbvie Inc, USA) surfactant of requiring smaller treatment volumes for a comparative clinical response. In this trial, poractant alfa is being used both for its established indication to treat RDS and as an effective carrying vehicle to deliver budesonide to the distal airways. The standard of care and comparator is poractant alfa alone (without a placebo added). We originally planned the use of an inert placebo, but this was declined by the HREC on the grounds it could potentially interfere with intrapulmonary surfactant distribution and compromise efficacy of poractant alfa in treating RDS. This is the rationale for not mixing a placebo with poractant alfa in the trial.

### Intervention description {11a}

#### Setting

The intervention will be performed in participating tertiary NICUs with the medications prepared in the NICU (or potentially in the delivery room in some centers) after the birth weight of the infant has been confirmed. Following randomization, the first intervention will be administered as soon as possible. If the infant meets the same treatment criteria 6–12 h after the first intervention, a second (and final) intervention will be administered in the NICU.

#### Preparation of the study intervention

To ensure treating clinicians are blinded, a two-person intervention team will be convened from on-duty staff who are not involved in the clinical care of the infant. Intervention teams will ideally be available 24 h/day and will consist of either a nurse and a doctor, two nurses, or a nurse/doctor and a pharmacist. The makeup of the intervention team may vary with the timing of the intervention (e.g., in-hours it is more likely that a pharmacist will be available) and by site. No members of the intervention team will be involved in data collection (other than data regarding the intervention preparation and administration itself) or outcome assessments for the study.

The study drugs will be made available in the NICU. Using the infant’s birth weight, the study medication will be prescribed by the clinical team and used by the intervention team to prepare the intervention in an area away from the infant and clinical team, where budesonide and surfactant (poractant alfa) are available. The intervention team will open the randomization envelope in a secure location and identify two further sealed mini envelopes labeled “Study ID: XX-X-XXX, Intervention 1” and “Study ID: XX-X-XXX, Intervention 2”. The intervention team will open the first (Intervention 1) envelope and draw up the correct dose (to the nearest 0.1 mL) of poractant alfa in a 3 mL (for infants with birth weight < 1000 g) or 5 mL (for infants with birth weight ≥ 1000 g) syringe. For infants allocated to surfactant plus budesonide, the dose of budesonide (to the nearest 0.01 mL) will be drawn up in a separate 1 mL syringe, then added to and mixed with poractant alfa by inverting the syringe several times. The two-person intervention team will complete appropriate double checks to ensure the doses drawn up are aligned with the prescription and the allocated treatment. To further maintain blinding, an opaque trial label will be applied around the syringe to hide the volume and appearance of the contents from bedside clinicians while it is being administered to the infant. The poractant alfa dose and the PLUSS Study ID will be charted on the infant’s prescription chart or electronic medical record for each intervention.

Member(s) of the intervention team will take the prepared intervention to the bedside of the enrolled infant. Before the allocated treatment is administered, pre-intervention patient observations will be documented in the peri-intervention case record form (CRF) by either the clinical or research team. Immediately prior to administration, the syringe will be hand-mixed by inverting it several times. An intervention team member will then either administer the prepared treatment when directed by the clinical team or hand the syringe to the clinical team to administer (depending on local administration protocols). The second mini envelope from the randomization envelope for Intervention 2 will be kept in a study pack, to be opened by the intervention team for the second intervention 6 to 12 h later if the infant is eligible.

In this pragmatic study, the following methods of intra-tracheal instillation will be permitted: standard bolus administration through an endotracheal tube (ETT) that will remain in situ with ongoing mechanical ventilation, INSURE (*in*tubate, *sur*factant, *e*xtubate) technique via an ETT, or via a thin catheter in those infants receiving non-invasive respiratory support (including CPAP, NIPPV or nasal high-flow) [39, 40].

Budesonide will be distributed to the NICU by the hospital pharmacy department and stored at room temperature. The poractant alfa used in the study will be accessed from ward stock. Stores of poractant alfa and budesonide will be maintained by the NICU pharmacist. Expiry dates for all medications will be clearly labeled and the pharmacist will be responsible for removing expired medications and replenishing stock.

Budesonide dosing for the PLUSS trial is based on the earlier study by Yeh and colleagues and evidence from pre-clinical studies [27, 32, 41, 42].

- Poractant alfa dose (both arms): 200 mg/kg initial dose; subsequent dose 100 mg/kg (if applicable)
- Budesonide dose (intervention arm): 0.25 mg/kg (0.5 mL/kg of 1 mg in 2 mL solution) added to each dose of poractant alfa*.*

### Criteria for discontinuing or modifying allocated interventions {11b}

**Fig. 1:**
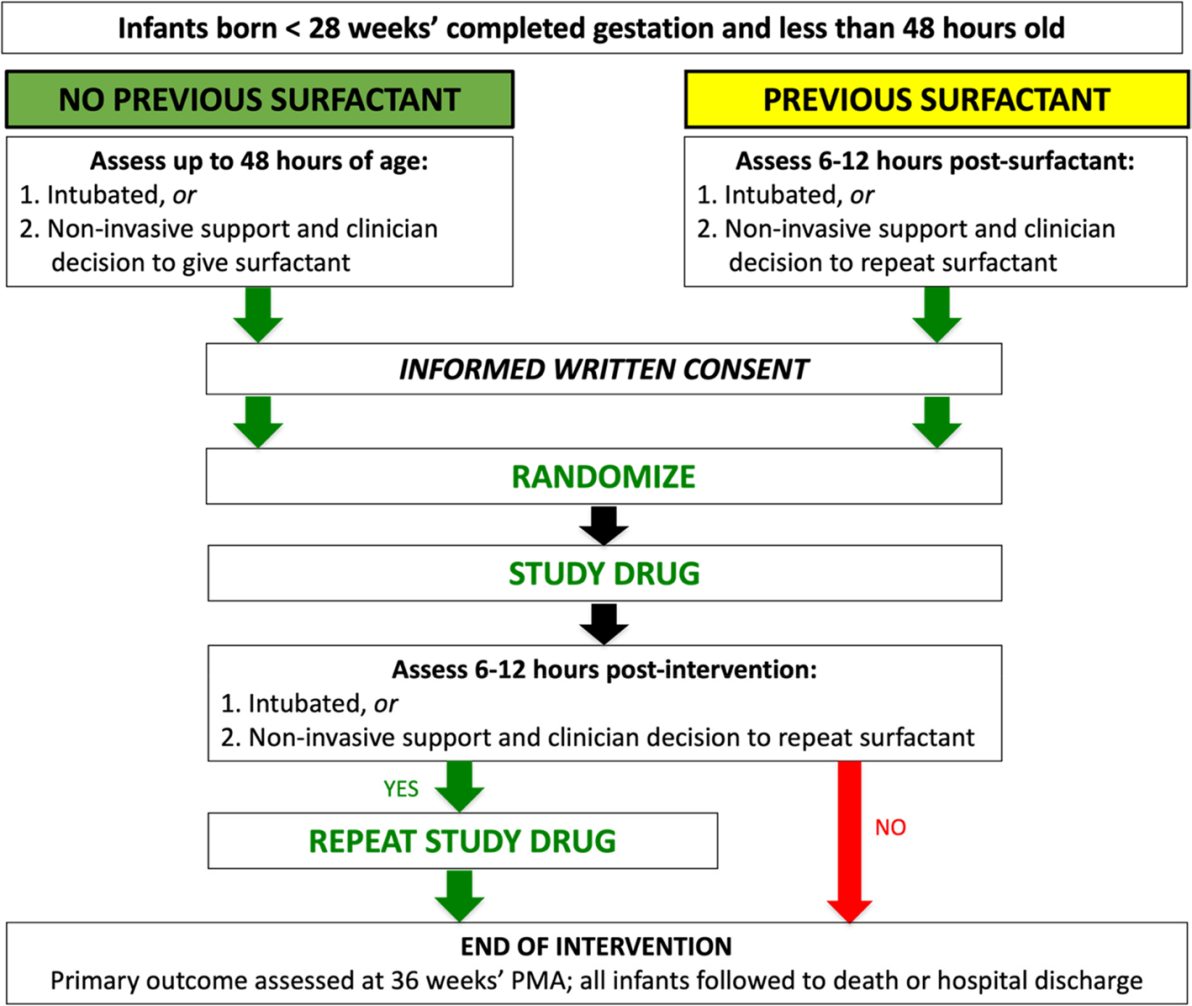
PLUSS In-Hospital Study Participant Flow Chart. PMA, post-menstrual age

Participants will receive a minimum of one and a maximum of two interventions (Fig. 1). Additional surfactant doses are at clinician discretion and outside the study protocol. Some infants enrolled in the trial may also have already received surfactant treatment prior to trial entry (Fig. 1). Adverse response to these interventions may be directly related to either exogenous surfactant or the investigational product, budesonide. Any adverse event attributed to the trial intervention, as assessed by the local site investigator, may lead to a protocol deviation if a second trial intervention is intentionally not given. The infant may, based on clinical response, undergo planned extubation from respiratory support prior to a second therapeutic intervention; this scenario will not be considered a protocol deviation. In addition, a parent may withdraw their child from further participation from the trial at any time.

### Strategies to improve adherence to interventions {11c}

Strategies to improve adherence to trial intervention protocols include intensive site initiation program followed by regular communication and support between the trial coordinating center and participating sites; regular monitoring of screening logs and data entry; continuing engagement with research nurses and site investigators at collaborators’ meetings; additional online trial meetings; site data auditing; regular trial newsletters; and having open lines of communication between the coordinating center and all participating sites/investigators.

### Relevant concomitant care permitted or prohibited during the trial {11d}

Inhaled or nebulized corticosteroid treatment will not be permitted in the trial until after the primary outcome has been determined (i.e., after 36 weeks’ PMA). All other management infants post randomization will be at the discretion of the clinical team. This includes escalating and weaning respiratory support, prescribing systemic (oral or intravenous) postnatal corticosteroids, oxygen saturation targeting, antibiotics for late-onset sepsis, enteral feeding, and diagnosis and treatment of a patent ductus arteriosus. Caffeine therapy is expected to be universal in this population. Oxygen saturation targets will be based on local unit guidelines.

### Provisions for post-trial care {30}

Data collection for the main trial outcomes ends either at discharge home from the primary hospital admission or death, whichever occurs first. Care during the primary hospitalization, if not specified in the trial protocol, will follow local practice guidelines. Similarly, post discharge care and support will adhere to local practice guidelines. In the event an infant suffers harm or injury as a result of trial participation, the trial coordinating center will liaise directly with the site investigators to ensure appropriate medical referrals are made and treatment provided. In Australia, infants eligible for Medicare will receive any medical treatment required to treat the injury or complication, free of charge, as a public patient in any Australian public hospital. Families retain all legal rights to obtain compensation for the injury.

### Outcomes {12}

#### Primary outcome

Rate of survival free of physiological BPD at 36 weeks’ PMA.

Physiological BPD will be assessed between 36^+0^ and 36^+6^ weeks’ PMA. Infants will be defined as having physiological BPD if any of the following criteria are met:

1. Receiving mechanical ventilation via an ETT, or positive pressure support, including CPAP, NIPPV, or nasal high flow ≥ 2 L/min, regardless of FiO_2_
*An effective FiO_2_ ≥ 0.30 if receiving supplemental ambient oxygen or via nasal prongs at < 2 L/min to maintain target oxygen saturations
*An effective FiO_2_ < 0.30 if receiving supplemental ambient oxygen or via nasal prongs at < 2 L/min to maintain target oxygen saturations AND an unsuccessful air reduction trial

*For infants receiving supplemental oxygen or nasal cannula oxygen, the effective FiO_2_ will be determined using the Benaron-Benitz formula [43].

The Trial Steering Committee has approved an algorithm for determining a diagnosis of BPD in cases where the BPD assessment is incorrectly or inadequately performed (e.g., wrong timing or incomplete data). This algorithm uses data collected at exactly 36^+0^ weeks’ PMA. In cases where the infant is receiving only supplemental oxygen or nasal prongs with gas flow < 2 L/min the effective FiO_2_ is calculated, and BPD is diagnosed if the effective FiO_2_ is $\ge$ 0.22, based on shared data from a recent large randomized trial [40].

#### In-hospital secondary outcomes (to be presented with the main trial results)

Secondary outcomes will be assessed from randomization up to primary discharge to home or 52 weeks’ PMA, whichever occurs sooner, unless otherwise specified.

1. Components of the composite primary outcome assessed at 36 + 0 to 36 + 6 weeks’ PMA:
  - Death before 36 weeks’ PMA*
  - Survival to 36 weeks’ PMA with physiological BPD
2. BPD severity/grade at 36 weeks’ PMA, as defined by Jensen et al. [44]
3. “Clinical BPD” at 40 + 0 weeks’ (term) PMA, defined as receiving any supplemental oxygen or any form of respiratory support (including mechanical ventilation, CPAP, NIPPV, or nasal high-flow)
4. Total deaths before hospital discharge and whether the death is classed as “respiratory” by the DSMB (see {22}); the age of death, mode of death, and categorization of the cause of death listed on the death certificate will be presented in a Supplementary Appendix
5. Treatment with postnatal systemic corticosteroids for lung disease
6. Severe brain injury on cranial ultrasound: severe (grade III or IV) [45, 46] intraventricular hemorrhage, and/or cystic periventricular leukomalacia
7. Severe (stage 2 or above) retinopathy of prematurity (ROP), as defined in the International Classification of ROP [46], and/or ROP treated with laser, cryotherapy, or intraocular therapy
8. Necrotizing enterocolitis, Modified Bell’s criteria stage 2 or greater [47]
9. Patent ductus arteriosus treated with anti-prostaglandin therapy or surgical ligation
10. Total duration of mechanical ventilation via an ETT in days
11. Discharge home on oxygen, or receiving any supplemental oxygen in hospital beyond 52 weeks’ PMA (whichever comes first)
12. Duration of positive pressure respiratory support (mechanical ventilation via an ETT, CPAP, NIPPV, nasal high-flow, or other positive pressure respiratory support) in days
13. Duration of supplemental oxygen in days
14. Length of hospital stay in days
15. Z-scores for weight, length, head circumference, and body mass index at 36 weeks’ PMA [48]

#### Adverse events (assessed from randomization until death, primary hospital discharge, or 52 week’s PMA, whichever occurs sooner, unless otherwise specified)

16. Spontaneous intestinal perforation (perforation not associated with necrotizing enterocolitis or other known pathology)*
17. The need for cardiopulmonary resuscitation (chest compressions) and/or administration of adrenaline/epinephrine (for resuscitation) within 24 h of the intervention*
18. Pneumothorax requiring drainage (needle thoracocentesis or intercostal catheter insertion)
19. Gastrointestinal hemorrhage, defined as fresh blood aspirated from an indwelling gastric tube, during the 14 days after the first intervention
20. Clinical diagnosis of pulmonary hemorrhage within the first 48 h after the first intervention
21. Any prescribed anti-hypertensive agents during the 14 days after the first intervention
22. Hyperglycemia > 10 mmol/L and/or receiving insulin therapy during the 14 days after the first intervention
23. Late-onset sepsis after 48 h of age, defined as a positive bacterial or fungal culture from a normally sterile site, or negative blood culture but clinical suspicion of sepsis and treatment with antibiotic/antifungal medication for ≥ 5 days)
24. Oral candidiasis during the first 14 days after the first intervention

*Defined as serious adverse events (SAEs).

#### Longer-term outcomes at 2 years and beyond (to be published separately to the main trial results)

At enrolment, parents/caregivers are informed of plans for longer-term follow-up of their child, and consent is obtained either for an assessment at 24 to 30 months’ corrected age or for future contact. Parents/caregivers who give consent for this 2-year follow-up/future contact will be asked to provide contact details. Contact will be maintained with families of surviving infants by sending birthday cards and/or trial newsletters, which will include a request to send updated contact details. A few months prior to a planned assessment, families will be reminded of this opportunity, and that an investigator will be in contact with them by phone and/or email beforehand to facilitate this. Assessments include a physical examination and health assessment (including hearing, vision, growth, and respiratory health); administration of the Bayley Scales of Infant and Toddler Development (3rd or 4th editions [49, 50]) for cognitive, language, and motor development; Gross Motor Function Classification System [51] to determine the severity of any cerebral palsy; and parent and child quality of life questionnaires using validated instruments. Funding has been obtained from Thrasher Research Fund (USA, www.thrasherresearch.org) and Cure Kids (New Zealand, www.curekids.org.nz) to ensure these assessments will be undertaken at all participating centers, and these outcome assessments have already begun in some centers. The results of 2-year outcome assessments will be published separately to the main trial outcomes.

The Primary outcome at a 2-year follow-up will be survival without moderate-severe neurodevelopmental disability, defined as any one or more of moderate or severe developmental delay, moderate or severe cerebral palsy, deafness, or blindness.

Secondary outcomes at 2 years will include:

- Death (from time of randomization)
- Age-standardized developmental scores for cognition, language, and motor function
- Developmental delay, defined as an age-standardized developmental score more than 1 standard deviation below the normative mean for cognition, language, or motor function
- Moderate or severe developmental delay, defined as an age-standardized developmental score more than 2 standard deviations below the normative mean for cognition, language, or motor function
- Cerebral palsy and severity (mild, moderate, severe, according to the Gross Motor Function Classification System)
- Moderate or severe neurosensory disabilities (moderate or severe cerebral palsy, blindness, and deafness)
- Sex- and age-standardized emotional-behavioral scores and proportion with emotional-behavioral difficulties
- Body size (sex- and corrected age-standardized scores for height, weight, head circumference, and body mass index)
- Incidence and severity of wheezing in the preceding 12 months
- Asthma risk score and proportion of children at high risk for school-age asthma
- Parent-reported child quality of life
- A cost-effectiveness analysis

In addition, we will describe the health-service utilization from initial hospital discharge until 2 years of age (corrected for prematurity), including emergency department presentations and inpatient hospitalizations (any cause and those related to a respiratory illness), and medication prescriptions for bronchodilators, inhaled corticosteroids, systemic corticosteroids, leukotriene antagonists and antibiotics where parents have provided consent for access to routine government datasets (e.g., Medicare and the Pharmaceutical Benefit Scheme in Australian centers).

Additional health outcome assessments are planned for later childhood and will be detailed in a separate protocol, subject to additional funding.

#### Cost-effectiveness analysis (to be published separately to the main trial results)

An economic evaluation assessing the cost-effectiveness of budesonide combined with surfactant compared with surfactant alone will be performed. A healthcare system perspective for cost will be adopted, and the time horizon will be the first 2 years of life. Costs being considered will include hospital and out-of-hospital care for the intervention and any adverse events. Effectiveness being considered will include survival free of BPD, and quality-adjusted life years. Extensive one-way and probabilistic sensitivity analyses will be conducted, which will include varying the cost perspective and incorporate cost to families, varying the time horizon to only include the initial hospitalization until discharge home or died, and excluding serious adverse events that were determined to be unrelated to trial participation as was determined by clinical assessment.

### Participant timeline {13}

**Table:** 

	**Enrolment**	**Allocation**	**Post-allocation**						**Post-discharge**
**Timepoint**	*Antenatal or postnatal (*< *48 h of age)*	*Within eligibility window* *(*< *48 h of age)*	< *48 h of age*	*6–59 h of age*	*28 days of age*	*36* + *0–36* + *6 weeks’ PMA (primary endpoint)*	*40 weeks’ PMA*	Hospital discharge*	*24–30 months’ (corrected for prematurity)*
**Enrolment**									
Eligibility screen	X								
Informed consent	X								
Maternal demographic and pregnancy data	X								
Randomization data		X							
Baseline infant data		X							
**Interventions**									
1st intervention			X						
2nd intervention (if applicable)				X					
**Outcome assessments**									
Immediate safety of intervention			Within 14 days after randomization						
Other in-hospital safety data			X	X	X	X	X	X	
Respiratory assessment day 28					X				
***Primary outcome: BPD assessment at 36 weeks’ PMA***						X			
“Clinical BPD” assessment at 40 weeks’ PMA							X		
Completion of admission data								X	
Longer-term outcome assessment									X

### Sample size {14}

The estimated incidence of the composite primary outcome of survival free of BPD is 50%, based on a review of data from the lead center (RWH) and published studies enrolling extremely preterm infants [18, 52, 53].

With a sample size of 1038 infants (519 in each group), the study has 90% power to detect a relative increase in survival free of BPD of 20% (absolute increase of 10%), from the anticipated event rate of 50% in the control arm to 60% in the intervention (budesonide) arm, alpha error 0.05. To allow for up to 2% study withdrawals or losses to follow-up, PLUSS will recruit a total of 1060 infants (530 in each arm).

### Recruitment {15}

Recruiting extremely preterm infants to highly powered RCTs always raises issues with feasibility due to the finite population of such infants who may be eligible. Our inclusion and exclusion criteria mean that most extremely preterm infants are eligible for PLUSS. In addition, infants remain eligible even if they have received one prior dose of surfactant. We have used our extensive national and international networks to identify and include 21 participating centers in 4 countries (Australia, New Zealand, Canada, Singapore) who regularly care for extremely preterm infants and have a strong research culture.

## Assignment of interventions: allocation

### Sequence generation {16a}

The randomization schedule is provided by the Clinical Epidemiology and Biostatistics Unit (CEBU) at the Murdoch Children’s Research Institute (MCRI), Melbourne, Australia. Randomization with balanced variable block sizes is used, stratified by study center, gestational age (22–25 weeks’ vs. 26–27 weeks’ completed gestation), prior surfactant therapy, and mode of respiratory support at randomization (mechanical ventilation via an endotracheal tube vs. non-invasive respiratory support).

### Concealment mechanism {16b}

When eligibility of an infant is confirmed, and prospective consent obtained, the infant is assigned to either receive surfactant plus budesonide, or surfactant alone, using a web-based randomization system with an allocation ratio of 1:1. A checklist on the website is used to confirm eligibility prior to randomization. Multiple births where more than one infant is eligible are randomized individually. A sealed opaque envelope at the study site is identified by the unique study ID generated from the web-based server (https://redcap.mcri.edu.au). The sealed envelope is opened by a dedicated intervention team who are not providing direct clinical care to the infant and will not be involved in any future outcome assessments. Inside the main envelope are a further two sealed envelopes for the first and second interventions respectively. Infants remain in their allocated group for repeat interventions (if applicable), with each envelope remaining sealed until the intervention team are ready to prepare the allocated medication.

### Implementation {16c}

Research staff at participating sites screen for eligible infants and approach families either antenatally or postnatally in the first 48 h for participation and to obtain informed consent. Once eligibility criteria are met and consent is obtained, research staff identify the two-person intervention team ensuring they are not involved in providing direct clinical care of the enrolled infant prior to randomization.

## Assignment of interventions: blinding

### Who will be blinded {17a}

Parents/caregivers, direct healthcare providers, outcome assessors, data analysts, and trial investigators are blinded to the randomization group.

Our experience is that it is virtually impossible to distinguish between the control and intervention study drugs, although there theoretically may be a subtle difference in the appearance of the surfactant with budesonide admixed. Additionally, the volume of the study drug to be administered is 0.5 mL/kg greater in the active treatment arm. To maintain blinding, the study drugs will be prepared by an independent intervention team (Sect. 2.9.2) whose members are not directly involved in the clinical care of the infant, and not involved in data collection or outcome assessments for the study. Data on the dose and type of intervention, as well as other data required by hospital pharmacies, will be recorded by the intervention team on allocation cards and stored in a secure lockbox only accessible by hospital pharmacists. In addition, after preparation of the intervention, the contents of the syringe will be covered using a stick-on label to obscure the volume and appearance to the bedside clinical staff.

The pharmacy departments of each participating center and CEBU will be the only other personnel aware of the allocated study intervention; they also will not be involved in data collection or outcome assessments for the study. Pharmacies will maintain a logbook of allocated study drugs and doses.

Neither the PLUSS Trial Steering Committee nor site researchers will be aware of the allocated interventions and will not be permitted access to this information until trial completion.

### Procedure for unblinding if needed {17b}

Unblinding before study end and database lock will not be permitted. Members of the DSMB will have access to unblinded treatment allocation to ascertain causality for any Serious Adverse Event (SAE, defined later) or other serious events that may be attributed to trial participation, and at pre-specified intervals for interim efficacy and safety analyses. At all times, the trial steering committee and site investigators will remain blinded, unless a site investigator is specifically requested to be unblinded when reporting a death or SAE by an independent assessor.

## Data collection and management

### Plans for assessment and collection of outcomes {18a}

Data collection will include outcomes previously described, as well as including the following screening and baseline data. This is not an exhaustive list of all data collected, and neither will all be reported in the final manuscript. A Statistical Analysis Plan will detail which baseline and screening data will be reported.

- Eligibility and randomization data: confirmed eligibility (meets all the inclusion criteria and none of the exclusion criteria), randomization date and time, study number
- Maternal data: age, parity, ethnicity, exposure to antenatal corticosteroids, treatment with magnesium sulfate, presence of chorioamnionitis (clinical and/or histological diagnosis), hypertensive disorders of pregnancy, prolonged rupture of membranes > 18 h, type of labor, mode of delivery
- Baseline infant data: date and time of birth, gestational age, sex, birth weight, multiplicity, treatments received in the delivery room, Apgar scores
- Pre-intervention data: treatment with surfactant (and dose and type), caffeine treatment, inotrope treatment, corticosteroids for hypotension, blood gas analysis results, most recent blood glucose concentration, fraction of inspired oxygen
- Intervention data (for first and second intervention(s)): date and time of intervention(s), method of intervention administration, clinical condition during and immediately after the intervention (episodes of bradycardia, oxygen desaturation, escalation of respiratory support level or resuscitation).

### Plans to promote participant retention and complete follow-up {18b}

We recognize for neonatal respiratory interventions that survival free of respiratory disease, normal lung function later in childhood, and longer-term (beyond infancy) development are important outcomes. However, we cannot incorporate outcomes beyond 2 years corrected age into the current trial protocol until additional funding is secured. We intend to maintain strong links with families through trial updates and birthday cards for infants enrolled to the study. Families will be notified of our intention to conduct longer-term health and development assessments and will have the opportunity to provide the research team consent in the future, to enable their child’s participation.

### Data management {19}

The PLUSS investigators at each site will be responsible for the collection of data which will be sourced from the medical notes of the mother and infant, parents, clinical staff, and bedside clinical charts. Paper CRFs and/or electronic data capture systems such as tablets or laptops at the patient’s cot-side will be used, and data will be entered into an electronic database (REDCap™, Vanderbilt University[54]) that will be designed and managed through CEBU. Completed CRFs will be checked for completeness and accuracy by researchers against the source data and verified by the trial data manager. All data will be securely stored for 25 years, and then securely destroyed/deleted.

### Confidentiality {27}

Participant data will be subject to data protection and privacy laws. All data will be securely stored, and electronic records will only be accessible by a password known to the research team. Parents and legal guardians also consent for their child’s health information to be linked with hospital data.

To maintain confidentiality, all data will be de-identified and stored in a password-protected electronic database and anonymity will be preserved in all scientific publications and presentations.

### Plans for collection, laboratory evaluation, and storage of biological specimens for genetic or molecular analysis in this trial/future use {33}

There will be no biological specimens collected.

## Statistical methods

### Statistical methods for primary and secondary outcomes {20a}

A detailed Statistical Analysis Plan will be finalized and submitted for publication prior to database lock. Data handling, verification, and analysis for the PLUSS trial will be performed by CEBU. Statistical analysis will follow standard methods for randomized trials, and reporting of findings will be performed in accordance with CONSORT guidelines.

The primary analysis will be by intention-to-treat. For dichotomous outcomes, including the primary outcome, the two treatment groups will be compared using risk difference with 95% CI, both overall, and within the pre-specified subgroups. The individual components of the primary outcome, death or physiological BPD at 36 weeks’ PMA, will be compared between the two treatment groups using risk difference with 95% CI, both overall, and within the pre-specified subgroups.

For dichotomous secondary outcomes, the two treatment groups will be compared using risk difference with 95% CI. For continuous outcomes, the two treatment groups will be compared using the difference of means, together with 95% CI, for outcome variables which are normally distributed; for outcome variables which are not normally distributed, the comparison will be the difference of medians, with 95% CI. Analyses of secondary outcomes will not be adjusted for multiple comparisons, but results will be interpreted cautiously.

All outcome differences between the two treatment groups will be estimated using regression models, with the randomization stratification factors included as covariates, and with standard errors adjusted to account for clustering due to multiple births. If there appears to be any imbalance in baseline prognostic factors, we may explore in secondary analyses the potential impact of any imbalance on the estimate of the exposure effect for the primary outcome.

Cost-effectiveness analysis will be conducted separately and reported following the CHEERS reporting guideline [55].

### Interim analyses {21b}

The DSMB have conducted multiple interim analyses for safety through the trial, and a single analysis for efficacy at the halfway point of recruitment when primary outcome endpoint data (death or BPD at 36 weeks’ PMA) were available for 530 infants. The DSMB reviewed safety outcomes after the primary outcome was known for 50, 100, 265 (25% planned recruitment), 530 (50%), and 800 (75%) infants. At all time-points the DSMB recommended that the trial continue unchanged.

### Methods for additional analyses (e.g., subgroup analyses) {20b}

For the primary outcome and its components subgroup analysis will be performed according to the randomization strata: gestational age, exposure to surfactant prior to randomization, and mode of respiratory support at randomization.

In addition, although we acknowledge that the trial is not powered for these analyses, we plan to assess the effect of important factors that might modulate the risk of death and BPD, including sex, small for gestational age, and the presence of chorioamnionitis.

### Methods in analysis to handle protocol non-adherence and any statistical methods to handle missing data {20c}

The primary analysis will be based on an intention to treat population accounting for all infants randomized. If necessary, multiple imputation methods will be used for missing data.

### Plans to give access to the full protocol, participant level-data and statistical code {31c}

In addition to full public access to clinical trial registration (ACTRN12617000322336; www.anzctr.org.au), information on the clinical trial is accessible at the PLUSS trial website (www.plusstrial.org).

## Oversight and monitoring

### Composition of the coordinating center and trial steering committee {5d}

The trial management team is based at The Royal Women’s Hospital (RWH), Melbourne, Australia, and meets weekly on average. The trial management team includes at least one of the Principal Investigators (BJM and COFK), the Data Manager and the Trial coordinator (JAD), the Trial Statistician (SD), and the New Zealand lead (CJM).

The Trial Steering Committee detailed below meets approximately quarterly, chaired by BJM.

Trial Steering Committee

**Table:** 

A/Prof Brett Manley (Co-Principal Investigator)	The Royal Women’s Hospital, Melbourne, Australia The University of Melbourne, Melbourne, Australia Murdoch Children’s Research Institute, Melbourne, Australia
Dr Omar Kamlin (Co-Principal Investigator)	The Royal Women’s Hospital, Melbourne, Australia The University of Melbourne, Melbourne, Australia Murdoch Children’s Research Institute, Melbourne, Australia
A/Prof Chris McKinlay	Department of Paediatrics: Child and Youth Health, The University of Auckland, Auckland, New Zealand
A/Prof Susan Jacobs	The Royal Women’s Hospital, Melbourne, Australia Murdoch Children’s Research Institute, Melbourne, Australia The University of Melbourne, Melbourne, Australia
Prof Lex Doyle	The Royal Women’s Hospital, Melbourne, Australia The University of Melbourne, Melbourne, Australia Murdoch Children’s Research Institute, Melbourne, Australia
Prof Peter Davis	The University of Melbourne, Melbourne, Australia Murdoch Children’s Research Institute, Melbourne, Australia
Prof Peter Dargaville	Royal Hobart Hospital, Hobart, Australia Menzies Institute for Medical Research, University of Tasmania, Hobart, Australia
Prof Jeanie Cheong	The Royal Women’s Hospital, Melbourne, Australia Murdoch Children’s Research Institute, Melbourne, Australia
A/Prof Susan Donath (Trial statistician)	Murdoch Children’s Research Institute, Melbourne, Australia
Dr Jennifer Dawson (Trial coordinator)	The Royal Women’s Hospital, Melbourne, Australia Murdoch Children’s Research Institute, Melbourne, Australia The University of Melbourne, Melbourne, Australia

Data and Safety Monitoring Board (DSMB)

**Table:** 

A/Prof David Cartwright	Chair	Brisbane, Australia
Prof Ian Marschner	Independent Statistician	Sydney, Australia
Prof Haresh Kirpalani	Independent Expert	Hamilton, Canada
Prof Brian Darlow	Independent Expert	Christchurch, New Zealand
Prof Rod Hunt	Independent Expert	Melbourne, Australia

### Composition of the Data Monitoring Safety Board (DSMB), its role and reporting structure {21a}

The DSMB has 5 independent members, comprising a Chair, 3 neonatal clinicians, and a biostatistician. The role of the DSMB was outlined in a DSMB Charter finalized prior to the trial commencing. The DSMB is responsible for safeguarding the interests of trial participants based on the accruing study data and the progress of the trial. Specifically, the DSMB will: (1) monitor and review participant safety in the trial; (2) monitor efficacy based on one pre-planned interim efficacy analysis at 50% of the planned sample size; (3) review participant recruitment, accrual, retention, and withdrawal; (4) monitor planned sample size assumptions; (5) advise the TSC if there is sufficient statistical evidence for a net clinical benefit or harm to warrant stopping the study early, and/or modifying other aspects of the study design to safeguard the interest of study patients while maintaining the scientific rigor of the study.

### Adverse event reporting and harms {22}

Safety reporting from the PLUSS Trial will follow standards from the 2016 recommendations of the National Health and Medical Research Council (NHMRC), Australia [56].

Pre-defined SAEs in the PLUSS trial are:

- Death (also a component of the primary outcome)
- Spontaneous intestinal perforation
- The need for cardiopulmonary resuscitation (chest compressions) and/or administration of adrenaline/epinephrine (for resuscitation) within 24 h of the intervention
- Any clinical deterioration of an infant requiring escalation of treatment that the treating clinician considers is secondary to the study intervention.

#### Reporting of adverse events and assessing their relatedness (causality) to trial interventions

The site principal investigator is responsible for reporting all pre-defined SAEs and any other unexpected but trial-related serious adverse events occurring from enrolment to hospital discharge to the Trial Coordinating Center within 7 calendar days of the investigator becoming aware of the event. All SAEs will be reviewed (blinded to group allocation) by the DSMB from clinical events summarized by the trial coordinator with the aim of completing this assessment within 7 working days of receiving the report. As death is a primary outcome of the PLUSS trial, an independent overview of all deaths is necessary with the DSMB assigning the likelihood of the death being related to the study intervention and whether the death was considered to be related to the infants’ lung disease (respiratory death). All SAEs (including deaths) will also have their relationship to the trial intervention assessed by the reporting site investigator with causality graded as “unrelated,” “possible,” “probable,” or “definite.”

### Frequency and plans for auditing trial conduct {23}

Trial conduct is repeatedly and continuously audited by the on-site independent pharmacist, and by the Trial Coordinator and Trial Data Manager. Auditing includes accuracy and completeness of randomization and intervention processes, reporting of protocol deviations by sites, and the accuracy and completeness of data entered into the electronic database. The Trial Data Manager continuously audits entered data and queries any missing data or potential inaccuracies. In addition, at the conclusion of the trial and prior to any statistical analysis, a selection of enrolled infants will have their source documents audited and compared to entered data.

### Plans for communicating important protocol amendments to relevant parties (e.g., trial participants, ethical committees) {25}

There have not been any major changes to the trial protocol since the trial began.

Minor changes and additions have been submitted for approval to The Royal Children’s Hospital Human Research Ethics Committee (Melbourne, Australia) and to all other relevant ethics committees, and then distributed and communicated to each participating site.

### Dissemination plans {31a}

Results will be communicated to those parents who requested to receive a trial report on the Consent Form. This will be via a provided email address and using lay language. The results of the trial will be presented at national and international conferences and the aim is to publish the main trial manuscript(s) in high-impact medical journals. Media and social media opportunities will be sought to communicate the results to the public.

## Discussion

Combining budesonide with surfactant for intratracheal administration is a simple intervention that may prevent BPD in extremely preterm infants and translate into health benefits in later childhood.

The international, randomized PLUSS trial is powered to allow the detection of an important difference in the primary outcome of survival free of BPD at 36 weeks’ PMA. The PLUSS trial will address gaps in the evidence for intratracheal budesonide due to its pragmatic and inclusive design, targeting all extremely preterm infants regardless of their initial mode of respiratory support.

Assessment of surviving infants at 2 years of age (corrected for prematurity) will provide evidence of longer-term efficacy and safety, which is critical for trials of corticosteroids in extremely preterm infants.

Should intratracheal budesonide mixed with surfactant increase survival free of BPD, without severe adverse effects, this readily available intervention could be introduced immediately into clinical practice, to the benefit of 100,000 s of extremely preterm infants and their families around the world.

## Trial status

The current trial protocol is version number 8, dated 14th July 2020. Recruitment began in January 2018 at RWH, Melbourne, with additional recruiting sites added over time. Recruitment is expected to be completed in early-mid 2023 with results expected in late 2023.

## Data Availability

The complete de-identified PLUSS trial dataset collected for this analysis will be available 6 months after publication of the primary outcome. This will include individual participant data. This study protocol and the trial Statistical Analysis Plan will also be available and will have been submitted for publication in a journal. The trial consent forms also will be available. An application to obtain the data from the MCRI may be made by emailing MCTC@mcri.edu.au. The final decision to share data will be made by the PLUSS Trial Steering Committee and the MCRI.

## Abbreviations

BPD: Bronchopulmonary dysplasia
CEBU: Clinical Epidemiology and Biostatistics Unit
CPAP: Continuous positive airway pressure
CRF: Case report form
DSMB: Data and Safety Monitoring Board
FiO_2_: Fraction of inspired oxygen
HREC: Human Research and Ethics Committee
NHMRC: National Health and Medical Research Council, Australia
NICU: Neonatal intensive care unit
NIPPV: Non-invasive positive pressure ventilation
PICF: Participant Information and Consent Form
PMA: Post-menstrual age
RDS: Respiratory distress syndrome
RWH: The Royal Women’s Hospital, Melbourne, Australia
SAE: Serious adverse event
SpO_2_: Peripheral oxygen saturation
